# Correction: Decorin inhibits glucose-induced lens epithelial cell apoptosis via suppressing p22^phox^-p38 MAPK signaling pathway

**DOI:** 10.1371/journal.pone.0354775

**Published:** 2026-07-28

**Authors:** Shanshan Du, Jingzhi Shao, Dandan Xie, Fengyan Zhang

After this article [1] was published, concerns were raised with Fig 1. Specifically, Figs 1A and 1D appear to overlap.

Co-first author JS stated that Fig 1D is incorrect. They provided an updated version of Fig 1 with the correct Fig 1D from the original experiments reported in [1]. The underlying data for Fig 1 are provided here in S1-S2 Files.

A member of the *PLOS One* Editorial Board reviewed the updated Fig 1 and stated that it supports the results in [1].

The raw data underlying Table 1 and Figs 1-8 are missing from the list of Supporting Information. The authors have provided the data as S2–S5 Files. With this correction, all relevant data are now provided.

**Fig 1:**
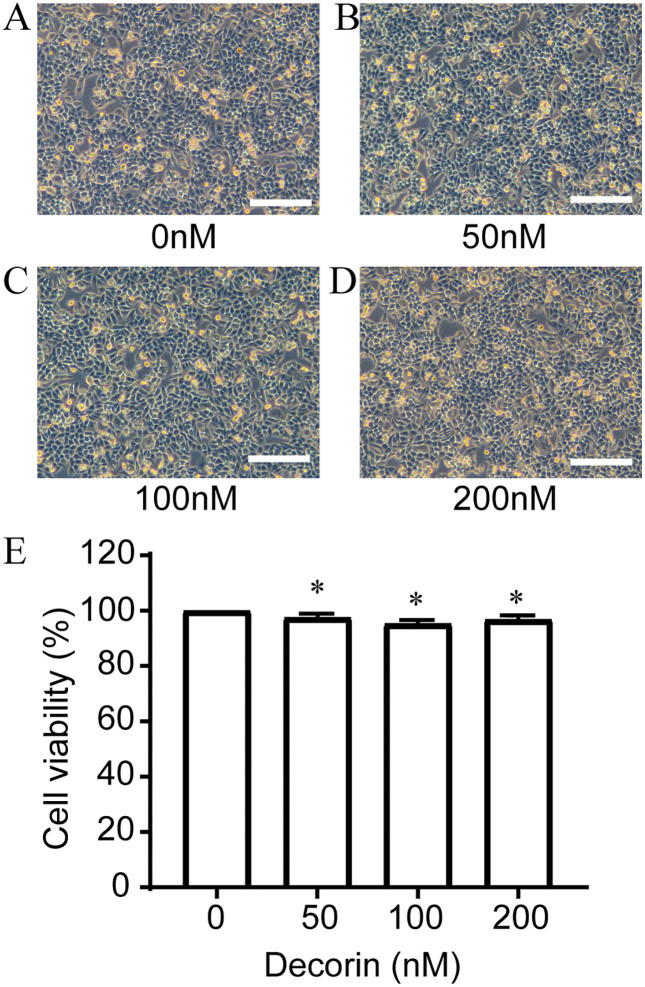
Cell viability of HLEB3 cells at different concentration of decorin as tested by CCK-8 assay. (A-D) Cell morphology at different concentrations of decorin. No obvious morphological change was found at different concentrations of decorin under the light microscope. (E) No significant difference in cell viability was found among the different concentration groups tested by CCK-8 assay (* *P* > 0.05 vs 0nM group). Bars are means ± SEM.

## Supporting information

S1 File Underlying images in support of Figs 1A-D. (ZIP)

S2 File Underlying individual-level data in support of Figs 1E, 2D, 3B-D, 4B, 4D, 5B, 5D, 5H, 6B-D, 7B, 7D, 8B and 8D. The underlying data for Figs 3B, 4B, 4D and 6B are raw values, whereas the corresponding graphs in [1] were generated using folds of these values against the control group.
(ZIP)

S3 File Underlying individual-level data in support of Fig 2B, and FACS output files in support of Figs 2A and 5C. (ZIP)

S4 File Underlying individual-level data in support of Fig 5F. (XLSX)

S5 File Underlying data in support of Table 1. (XLSX)
