# Supplementary figures and images for: Correction: Decorin inhibits glucose-induced lens epithelial cell apoptosis via suppressing p22phox-p38 MAPK signaling pathway
Source: PLoS One. 2026 Jul 28;21(7):e0354775. doi: 10.1371/journal.pone.0354775 (PMC13411911; doi:10.1371/journal.pone.0354775)

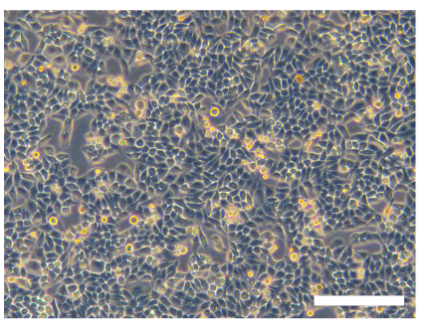

Supplement: S1 File — (ZIP) [file pone.0354775.s001.zip › Fig1 data/Fig1A-0nM.png]

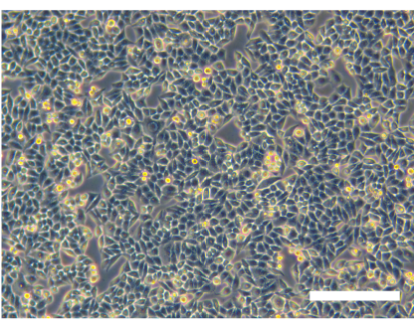

Supplement: S1 File — (ZIP) [file pone.0354775.s001.zip › Fig1 data/Fig1B-50nM.png]

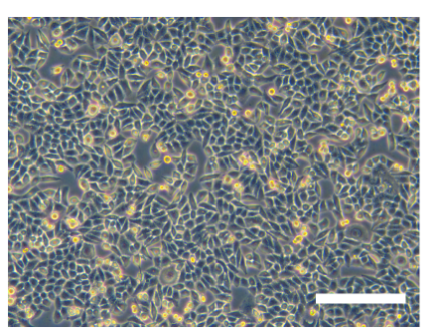

Supplement: S1 File — (ZIP) [file pone.0354775.s001.zip › Fig1 data/Fig1C-100nM.png]

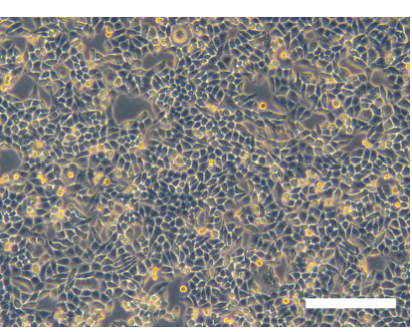

Supplement: S1 File — (ZIP) [file pone.0354775.s001.zip › Fig1 data/Fig1D-200nM.png]

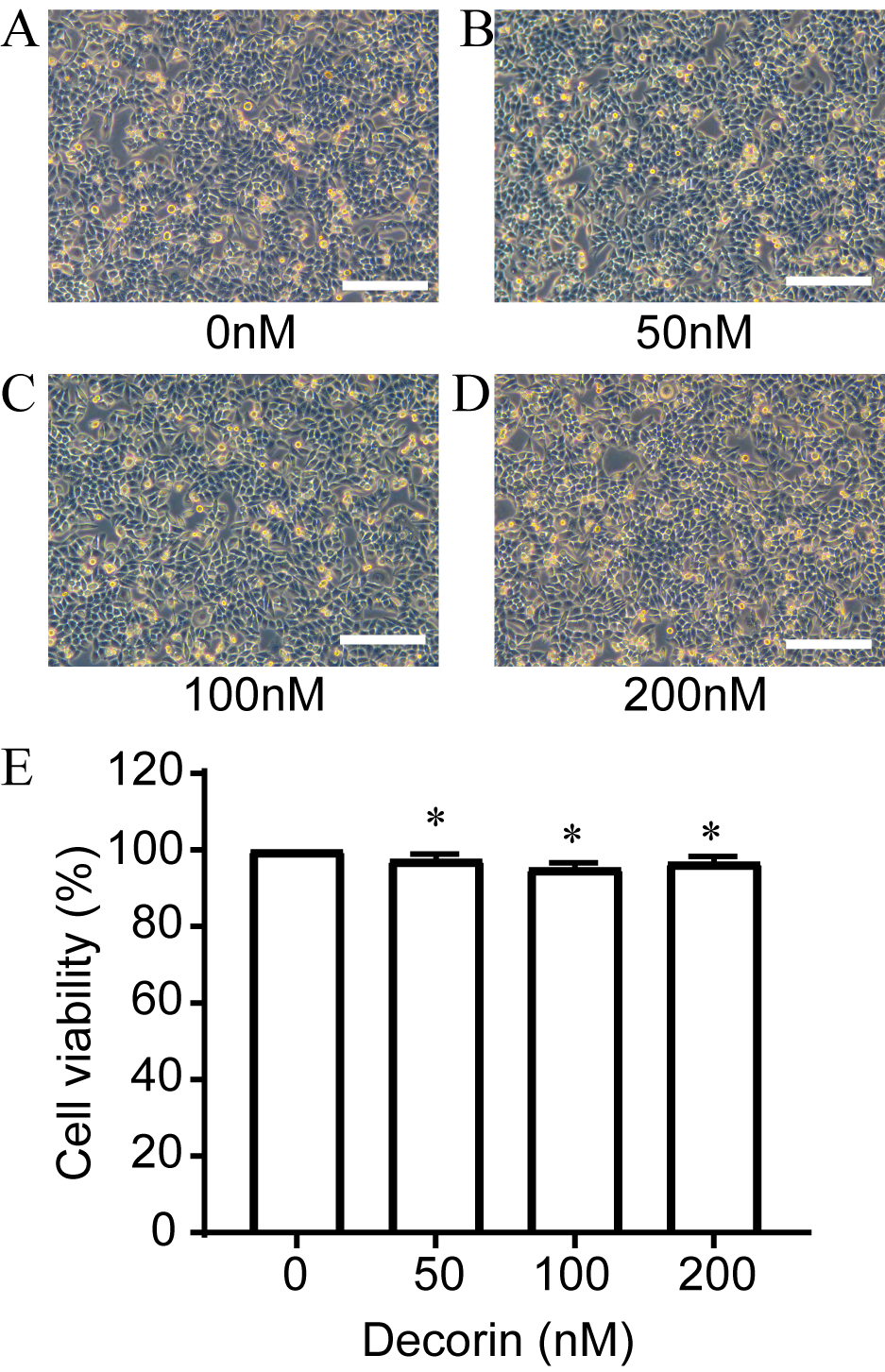

Supplement: S1 File — (ZIP) [file pone.0354775.s001.zip › Fig1 data/fig1(revised).tif]

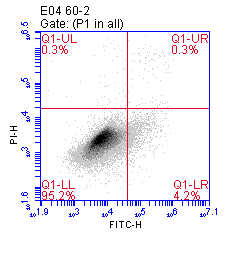

Supplement: S2 File — The underlying data for Figs 3B, 4B, 4D and 6B are raw values, whereas the corresponding graphs in [1] were generated using folds of these values against the control group. (ZIP) [file pone.0354775.s002.zip › Fig data/Fig2 data/Fig2A/Con 4.2.png]

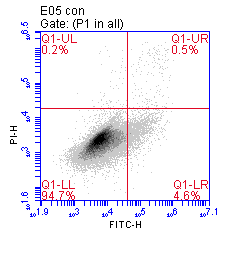

Supplement: S2 File — The underlying data for Figs 3B, 4B, 4D and 6B are raw values, whereas the corresponding graphs in [1] were generated using folds of these values against the control group. (ZIP) [file pone.0354775.s002.zip › Fig data/Fig2 data/Fig2A/HD 4.6.png]

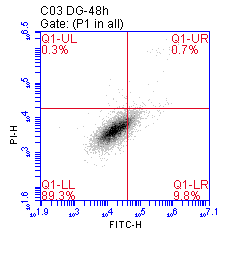

Supplement: S2 File — The underlying data for Figs 3B, 4B, 4D and 6B are raw values, whereas the corresponding graphs in [1] were generated using folds of these values against the control group. (ZIP) [file pone.0354775.s002.zip › Fig data/Fig2 data/Fig2A/HG 9.8.png]

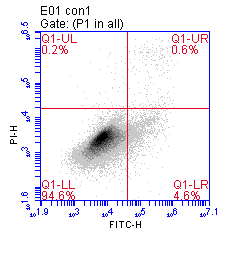

Supplement: S2 File — The underlying data for Figs 3B, 4B, 4D and 6B are raw values, whereas the corresponding graphs in [1] were generated using folds of these values against the control group. (ZIP) [file pone.0354775.s002.zip › Fig data/Fig2 data/Fig2A/Mannitol 4.6.png]

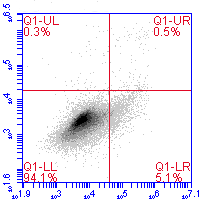

Supplement: S2 File — The underlying data for Figs 3B, 4B, 4D and 6B are raw values, whereas the corresponding graphs in [1] were generated using folds of these values against the control group. (ZIP) [file pone.0354775.s002.zip › Fig data/Fig5 data/Fig5C/Con 5.1-3cm.tif]

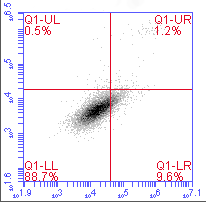

Supplement: S2 File — The underlying data for Figs 3B, 4B, 4D and 6B are raw values, whereas the corresponding graphs in [1] were generated using folds of these values against the control group. (ZIP) [file pone.0354775.s002.zip › Fig data/Fig5 data/Fig5C/HG 9.6-3cm.tif]

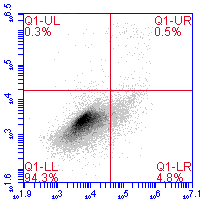

Supplement: S2 File — The underlying data for Figs 3B, 4B, 4D and 6B are raw values, whereas the corresponding graphs in [1] were generated using folds of these values against the control group. (ZIP) [file pone.0354775.s002.zip › Fig data/Fig5 data/Fig5C/HG+ siRNA 4.8-3cm.tif]

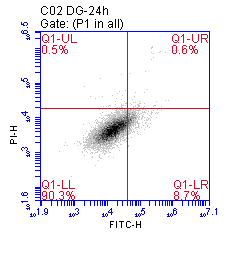

Supplement: S2 File — The underlying data for Figs 3B, 4B, 4D and 6B are raw values, whereas the corresponding graphs in [1] were generated using folds of these values against the control group. (ZIP) [file pone.0354775.s002.zip › Fig data/Fig5 data/Fig5C/HG+con siRNA 8.7.png]
